# Flexible negative-pressure ureteral sheath lithotripsy compared with mini-PCNL for upper urinary tract stones with moderate-to-severe hydronephrosis: a retrospective clinical study

**DOI:** 10.1186/s12894-026-02115-3

**Published:** 2026-03-19

**Authors:** Wei Hao, Junxiu Hao, Min Zhang, Huimin Zhang, Chao Yang

**Affiliations:** Department of urology, Tangshan Gongren Hospital, 27 Wenhua Road, Lubei District, Tangshan, Hebei Province 063000 China

**Keywords:** Flexible negative-pressure ureteral sheath lithotripsy (FANS), Mini-percutaneous nephrolithotomy (mini-PCNL), Moderate-to-severe hydronephrosis, Upper urinary tract stones

## Abstract

**Background:**

Management of upper urinary tract stones with moderate-to-severe hydronephrosis remains challenging due to altered renal anatomy and higher complication risks. Flexible negative-pressure ureteral sheath lithotripsy (FANS) has emerged as a minimally invasive alternative to miniaturized percutaneous nephrolithotomy (mini-PCNL). This study compared the efficacy and safety of FANS and mini-PCNL in this patient population.

**Methods:**

A total of 125 patients with upper urinary tract stones and moderate-to-severe hydronephrosis who underwent either FANS (*n* = 57) or mini-PCNL (*n* = 68) between June 2024 and June 2025 were retrospectively analyzed. Baseline demographics, stone characteristics, operative parameters, perioperative laboratory changes, hospital stay, immediate stone-free rate (SFR) and finial SFR, and postoperative complications were assessed.

**Results:**

Baseline characteristics (age, BMI, stone burden, comorbidities, and hydronephrosis degree) were comparable between groups. The FANS group had a longer operative time (70 vs. 60 min, *P* = 0.006) but a shorter stay of hospital (2 vs. 3 days, *P* = 0.008) and smaller hemoglobin drop (8.0 vs. 11.0 g/L, *P* = 0.031). WBC changes, eGFR drop, immediate SFR (80.7% vs. 80.9%) and finial SFR (89.5% (51/57) vs. 88.2% (60/68)) were similar. The overall complication rate was lower with FANS (5.9% (4/57) vs. 25.0% (17/68), *P* = 0.011), and no severe complications occurred, whereas mini-PCNL included several Grade 3b events.

**Conclusions:**

Both FANS and mini-PCNL achieved comparable stone clearance in moderate-to-severe hydronephrosis. However, FANS offered reduced bleeding, faster recovery, and fewer complications, indicating its potential role as a safer and less invasive option for patients with complex upper urinary tract anatomy.

## Background

Upper urinary tract stones complicated by moderate-to-severe hydronephrosis present unique surgical challenges. Percutaneous nephrolithotomy (PCNL) has long been recognized as the gold standard for the management of large renal stones, particularly those exceeding 20 mm in diameter. The high stone-free rate (SFR) associated with PCNL have made it the preferred approach for complex or staghorn calculi, and it allows for direct access to the renal collecting system for efficient stone fragmentation and removal [1, 2]. However, conventional PCNL is associated with notable risks, including significant intraoperative bleeding, injury to surrounding structures, and prolonged hospital stay, particularly in patients with altered renal anatomy or comorbidities [3, 4]. In response to the need for less invasive procedures, miniaturized PCNL (mini-PCNL) was developed. By utilizing smaller access sheaths (typically 14–20 Fr), mini-PCNL reduces parenchymal trauma, decreases intraoperative blood loss, and lowers postoperative pain while maintaining comparable stone-free rates to standard PCNL [5, 6].

Flexible ureteroscopy (FURS) has become a widely adopted endourological approach and is recommended as a first-line treatment for most renal stones smaller than 2 cm according to contemporary guidelines. In selected patients, it may also be considered for larger or more complex stones, particularly when patient factors or anatomical considerations favor a retrograde approach [7, 8]. While FURS is minimally invasive and allows for precise intrarenal visualization, its application in larger stones has been limited by longer operative times, incomplete fragment removal, and increased intrarenal pressure, which may elevate the risk of postoperative infectious complications [9].To overcome these limitations, Flexible negative-pressure ureteral sheath lithotripsy (FANS) has recently been introduced. FANS integrates a flexible ureteroscope with a negative-pressure suction system, allowing continuous aspiration of stone fragments and debris, potentially reducing intrarenal pressure, and minimizing the risk of infection or postoperative renal injury [10–12].

Comparative analyses of mini-PCNL and FANS in the general population of patients with renal stones have indicated that both approaches can achieve satisfactory stone clearance, with FANS offering potential advantages in terms of reduced intrarenal pressure and lower perioperative morbidity [10, 13]. However, data specifically addressing patients with moderate-to-severe hydronephrosis are limited. Moderate-to-severe hydronephrosis, characterized by significant dilation of the renal pelvis and calyces, often coexists with upper urinary tract stones. This condition reflects obstructed urine drainage, typically due to pelvis-ureteric junction or upper ureteral obstruction, resulting in increased renal pelvic pressure. Chronic hydronephrosis can compress renal parenchyma, leading to cortical thinning, tubular atrophy, and irreversible loss of renal function [14]. The presence of severe hydronephrosis may also reduce the stone-free rate and predispose patients to higher complication rates postoperatively [15].

Despite the increasing adoption of mini-PCNL and FANS in clinical practice, there remains a lack of direct comparative evidence for their use specifically in patients with moderate-to-severe hydronephrosis. This represents a significant knowledge gap, as these patients are at higher risk for surgical complications due to altered renal anatomy, increased intrarenal pressure, and compromised renal parenchyma. Understanding the relative efficacy and safety of mini-PCNL versus FANS in this high-risk population is therefore crucial to inform optimal surgical decision-making. Accordingly, the present study was designed to retrospectively compare mini-PCNL and FANS in patients with moderate-to-severe hydronephrosis and upper urinary tract stones, with key outcomes including SFR, operative time, perioperative complications, and postoperative renal function recovery. This study aimed to provide clinical evidence to support individualized treatment selection in this challenging population.

## Methods

### Study design and ethical approval

This retrospective cohort study was conducted at Tangshan Gongren Hospital between June 2024 and June 2025. The Ethics Committee agreed the information and data for clinical research. Informed consent was obtained from the patients for collecting their data (2024KY045).

### Patient selection

Medical records of patients diagnosed with upper urinary tract stones complicated by moderate-to-severe hydronephrosis were reviewed.

Inclusion criteria: (1) Age ≥ 18 years; (2) Radiologically confirmed upper urinary tract stones with moderate-to-severe hydronephrosis (e.g., Society for Fetal Urology grade 3–4 or renal pelvis diameter >20 mm [16]); (3) Underwent either mini-PCNL or FANS; (4) Complete preoperative and postoperative clinical and imaging data available.

Exclusion criteria: (1) Active urinary tract infection not controlled by antibiotics; (2) Coagulopathy or bleeding disorders; (3) Pregnancy; (4) Severe cardiopulmonary comorbidities; (5) Previous ipsilateral renal surgery within 6 months.

### Treatment allocation

Treatment allocation was determined according to structured clinical assessment rather than arbitrary preference. The degree of hydronephrosis and stone size were not the primary determinants of procedural selection.

FANS was generally preferred in patients with relative contraindications to percutaneous renal access, such as coagulopathy, increased bleeding risk, or spinal deformity. Mini-PCNL was primarily selected for complex lower calyceal stones, especially when the infundibulopelvic angle was narrow or when concomitant ureteropelvic junction obstruction required simultaneous management. Final decisions were made based on anatomical feasibility and overall surgical safety.

### Surgical procedures

All mini-PCNL procedures were performed under general anesthesia. Initially, a Fr6 open-ended ureteral catheter was inserted with the patient in the lithotomy position to facilitate intraoperative identification of the collecting system and ensure drainage. The patient was then repositioned to the prone position for percutaneous access. Percutaneous renal puncture was guided by ultrasound, targeting a calyx appropriate for maximal stone access. A guidewire was introduced into the collecting system, followed by sequential tract dilation using metal or fascial dilators to establish a 14–16 Fr percutaneous tract. Stone fragmentation was achieved using holmium: YAG laser (200–365 μm fiber, 1.0–1.5 J, 15–30 Hz), depending on stone composition and size. Fragments were removed through the through the nephroscope. At the end of the procedure, a double-J ureteral stent 6 Fr was routinely placed to maintain ureteral patency, a nephrostomy tube (12–14 Fr) or tubeless (defined as omission of the nephrostomy tube while maintaining ureteral drainage with a stent) approach was applied based on intraoperative findings and bleeding. The operative time, intraoperative complications, and estimated blood loss were recorded.

**Fig. 1:**
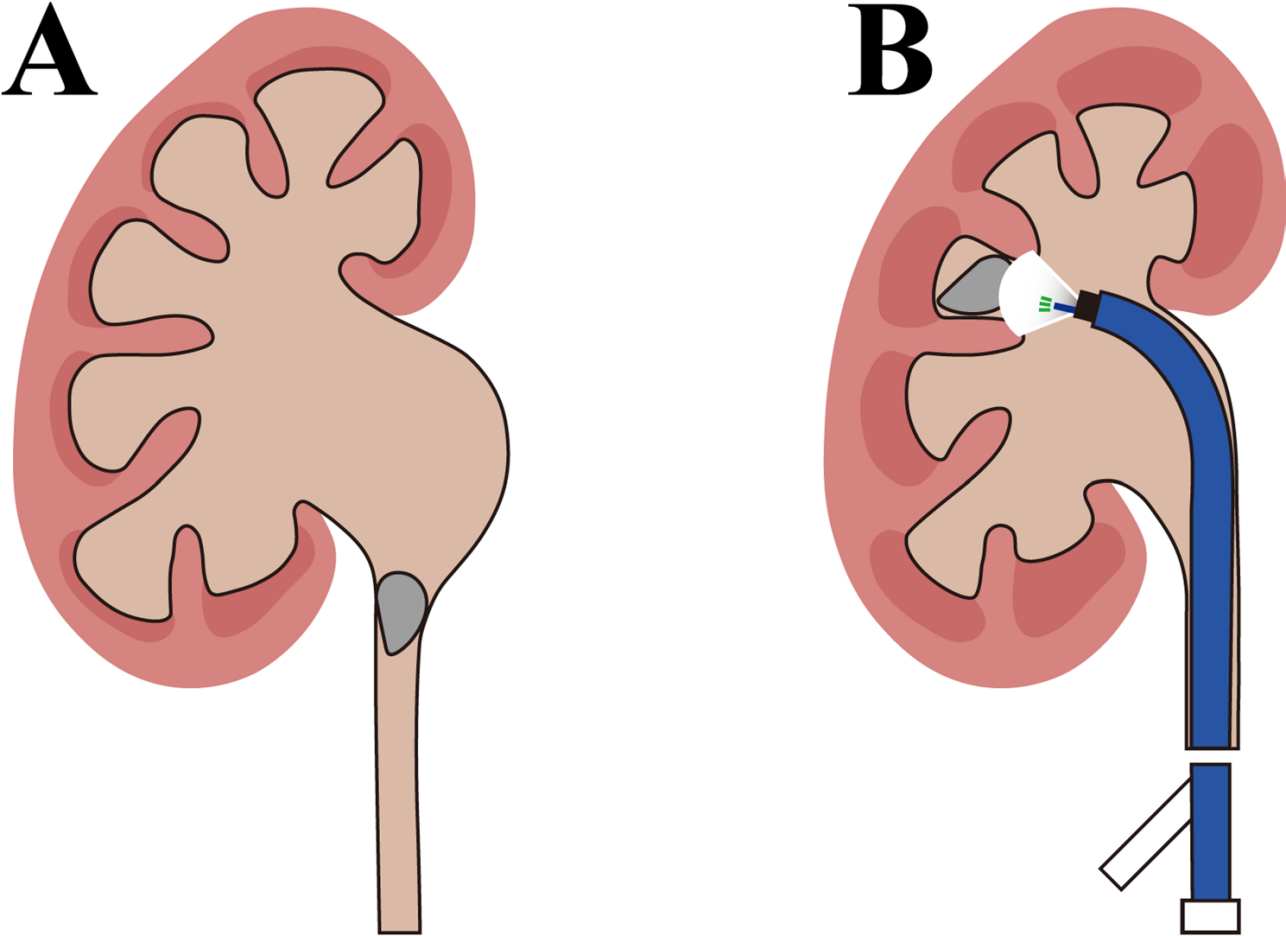
Schematic illustration of moderate-to-severe hydronephrosis with renal calculi before and after treatment. FANS before (**A**) and during treatment (**B)**

All FANS procedures were performed under general anesthesia. The patient was placed in the lithotomy position. A hydrophilic guidewire was first introduced under fluoroscopic guidance, and a semi-rigid ureteroscope (if ureteral stones were present) was advanced to the site of the ureteral stone. When ureteral stones were encountered, they were gently pushed into the renal pelvis whenever feasible to facilitate subsequent lithotripsy. The guidewire was left in place to maintain access. A negative-pressure ureteral access sheath (12/14 Fr, 35–45 cm) (Copper Medical Technology Co., Ltd.) was then advanced over the guidewire into the ureter. A flexible ureteroscope (Shanghai Innovex Medical Devices Co., Ltd.) was inserted through the sheath to visualize the renal collecting system. Stone fragmentation was performed using a holmium: YAG laser (200 μm fiber, 0.6–1.0 J, 10–30 Hz) with dusting or fragmentation settings depending on stone size and composition. A continuous negative-pressure suction system connected to the ureteral sheath aspirated stone fragments and dust, reducing intrarenal pressure and minimizing postoperative infection risk. Stone fragments were removed using basket forceps or suction, ensuring maximal clearance of debris (Fig. 1). At the end of the procedure, a double-J ureteral stent 6 Fr was routinely placed to maintain ureteral patency. Operative time, intraoperative complications, and estimated blood loss was recorded.

### Outcome measures

Immediate SFR and finial SFR (three months postoperative) were recorded, and was graded according to CT-based criteria as: Grade A, no residual stones; Grade B, residual fragments < 2 mm; Grade C, residual fragments 2–3.9 mm; and Grade D, residual fragments ≥ 4 mm [17]. Operative parameters included operative time and estimated intraoperative blood loss. Perioperative laboratory changes comprised postoperative hemoglobin levels, hemoglobin drop, white blood cell (WBC) count change, and estimated glomerular filtration rate (eGFR) drop, eGFR was retrospectively calculated using the CKD-EPI creatinine Eq. (2009) based on serum creatinine, age, and sex [18]. Preoperative laboratory values were obtained within 48 h prior to surgery, and postoperative measurements were performed 1 h after completion of the procedure. Hospitalization outcomes included length of hospital stay and 30-day readmission rate. Stone characteristics were analyzed in terms of maximum diameter, volume, density (Hounsfield units), and composition (calcium oxalate monohydrate, calcium oxalate dihydrate, calcium phosphate, calcium carbonate, and uric acid). Postoperative complications were recorded and graded according to the Clavien–Dindo classification [19].

### Statistical analysis

Continuous variables were expressed as mean ± standard deviation (SD) or median (range), depending on data distribution, and compared between groups using independent t-tests for normally distributed data or Mann–Whitney U tests for non-normally distributed data. Categorical variables were presented as counts and percentages, and analyzed using the chi-square test or Fisher’s exact test, as appropriate. A two-sided p-value < 0.05 was considered statistically significant. All analyses were performed using SPSS version 26.0 (IBM Corp., Armonk, NY, USA).

## Results

### Patient demographics and preoperative characteristics

A total of 125 patients were included in this study, comprising 57 patients in the FANS group and 68 patients in the mini-PCNL group (Table 1). The median age was comparable between groups (58 years (28–77) vs. 58.5 years (32–84), *p* = 0.922). Body mass index (BMI) was also similar (25.8 (19.5–44.1) vs. 25.5 (19.1–35.3), *p* = 0.794). Stone burden parameters, including maximum stone diameter (21.0 mm (11.5–30.0) vs. 20.0 mm (10.0–30.0), *p* = 0.253), stone volume (1994.7 mm³ (328.3–7388.0) vs. 1689.8 mm³ (153.8–9093.1), *p* = 0.327), and stone density (953.5 HU (320.0–1338.0) vs. 988.8 HU (286.0–1783.0), *p* = 0.240), showed no statistically significant differences. Baseline renal function and hematologic parameters were balanced between groups. Regarding comorbidities, the prevalence of hypertension (45.6% (26/57) vs. 42.6% (29/68), *p* = 0.857) and diabetes mellitus (28.1% (16/57) vs. 25.0% (17/68), *p* = 0.839) did not differ significantly between groups. Gender distribution was balanced (male: 57.9% (33/57) vs. 58.8% (40/68), *p* = 1.000), as was side of stones (right: 52.6% (30/57) vs. 52.9% (36/68), *p* = 1.000). Preoperative ureteral stent placement was performed in 38.6% (22/57) of FANS cases and 30.9% (21/68) of mini-PCNL cases (*p* = 0.450). The degree of hydronephrosis was comparable between groups, with moderate hydronephrosis present in 61.4% (35/57) vs. 54.4% (37/68), and severe hydronephrosis in 38.6% (22/57) vs. 45.6% (31/68) (*p* = 0.471). Overall, there were no significant differences in baseline demographics, comorbidities, stone characteristics, or preoperative laboratory findings between the two groups, indicating good comparability of study populations.

**Table 1:** Patient demographics and preoperative findings

Variable	FANS (57)	Mini-PCNL (68)	*P* value
Medial (Min–Max)			
Age, year	58(28–77)	58.5(32–84)	0.922
BMI, kg/m^2^	25.8(19.5–44.1)	25.5(19.1–35.3)	0.794
Stone size, mm	21.0(11.5–30.0)	20.0(10.-30.0)	0.253
Stone volume, mm^3^	1994.7(328.3–7388.0)	1689.8(153.8-9093.1)	0.327
Stone density, HU	953.5(320.0-1338.0)	988.8(286.0-1783.0)	0.240
Pre-eGFR, ml/min/1.73 m²	88.8 (14.8–137.3)	81.2 (19.5–117.5)	0.052
Pre-hemoglobin, g/L	130(94–192)	134(85–166)	0.214
White blood cells, *10^9^/L	6.8(4.15–10.53)	6.3(3.3–12.0)	0.535
White urine cells, N/HPF	14.6(0.0-82.8)	16.7(0.0-391.0)	0.414
N (%)			
Hypertension	26 (45.6)	29 (42.6)	0.857
Diabetes mellitus	16 (28.1)	17 (25.0)	0.839
Gender			
Male	33 (57.9)	40 (58.8)	1.000
Female	24 (42.1)	28 (41.2)	
Side			
Right	30 (52.6)	36 (52.9)	1.000
Left	27 (47.4)	32 (47.1)	
Preoperative stent placement			
No	35 (61.4)	47 (69.1)	0.450
Yes	22 (38.6)	21 (30.9)	
Hydronephrosis			
Moderate	35 (61.4)	37 (54.4)	0.471
Severe	22 (38.6)	31 (45.6)	

*FNAS* Flexible negative-pressure ureteral sheath lithotripsy, *PCNL* Percutaneous nephrolithotomy, *BMI* Body mass index, *N* Number

### Perioperative and postoperative outcomes

Perioperative and postoperative outcomes are summarized in Table 2. The median operative time was significantly longer in the FANS group compared with the mini-PCNL group (70.0 min (30.0–139.0) vs. 60.0 min (30.0–112.0), *P* = 0.006). The postoperative hemoglobin level was comparable between the two groups (120.0 g/L (91.0–176.0) vs. 120.5 g/L (79.0–161.0), *P* = 0.378). However, the hemoglobin drop was significantly lower in the FANS group (8.0 g/L (0.0–19.0)) than in the mini-PCNL group (11.0 g/L (2.0–34.0), *P* = 0.031). No significant differences were observed in postoperative eGFR (82.5 ml/min/1.73m^3^ vs. 74.0 ml/min/1.73m^3^, *P* = 0.051) or eGFR drop (5.5 ml/min/1.73m^3^ vs. 4.8 ml/min/1.73m^3^, *P* = 0.995). Similarly, postoperative WBC and WBC rise did not differ between the groups (*P* = 0.327 for both). The length of hospital stay was shorter in the FANS group compared with mini-PCNL (2 days (2–5) vs. 3 days (2–7), *P* = 0.008).

**Table 2:** Comparison of outcomes between the two groups

Variable	FANS (57)	Mini-PCNL (68)	*P* value
Medial (Min–Max)			
OR time, min	70.0(30.0-139.0)	60.0(30.0-112.0)	0.006
Post-hemoglobin, g/L	120.0(91.0-176.0)	120.5(79.0-161.0)	0.378
Hemoglobin drop, g/L	8.0(0.0–19.0)	11.0(2.0–34.0)	0.031
Post-eGFR, ml/min/1.73 m²	82.5 (14.4–131.8)	74.0 (18.9–114.6)	0.051
eGFR drop, ml/min/1.73 m²	5.5 (-7.1–22.4)	4.8 (0.6–11.7)	0.995
Postoperative WBC, *10^9^ /L	9.08(3.28–18.78)	8.45(3.21–20.75)	0.327
WBC rise, *10^9^ /L	2.84(-3.15-11.83)	2.27(-4.64-14.46)	0.327
Length of hospital stay, day	2(2–5)	3(2–7)	0.008
N (%)			
Immediate SFR			
Total < 4 mm	46 (80.7)	55 (80.9)	1.000
0 mm	21 (36.8)	32 (47.1)	0.279
> 0 to < 2 mm	5 (8.8)	8 (11.8)	0.770
2 to 3.9 mm	20 (35.1)	15 (22.1)	0.115
Final SFR			
Total < 4 mm	51 (89.5)	60 (88.2)	1.000
0 mm	41 (71.9)	45 (66.2)	0.563
> 0 to < 2 mm	0 (0.0)	0 (0.0)	-
2 to 3.9 mm	10 (17.5)	15 (22.1)	0.607
Re-treatment			0.742
FURS for calyceal stones	1 (1.8)	1 (1.5)	
ESWL	2 (3.5)	2 (2.9)	
stone composition			0.073
COD	19 (33.3)	39 (57.4)	
COM	28 (49.1)	20 (29.4)	
CP	5 (8.8)	4 (5.9)	
CC	0 (0.0)	1 (1.5)	
﻿ UA	5 (8.8)	4 (5.9)	

*FNAS* Flexible negative-pressure ureteral sheath lithotripsy, *PCNL* Percutaneous nephrolithotomy, *OR* Operative, *WBC* White blood cell, *N* Number, *FURS* Flexible ureteroscopy, *ESWL* Extracorporeal shock wave lithotripsy, *COD* Calcium oxalate dihydrate, *COM* Calcium oxalate monohydrate, *CP* Calcium phosphate, *CC* Cystine calculus, *UA* Uric acid

Regarding immediate SFR, the overall SFR (fragments < 4 mm) was similar between the two groups (80.7% (46/57) vs. 80.9% (55/68), *P* = 1.000). Subgroup analysis showed no significant differences among complete clearance (36.8% (21/57) vs. 47.1% (32/68), *P* = 0.279), residual fragments 0–2 mm (8.8% (5/57) vs. 11.8% (8/68), *P* = 0.770), and residual fragments 2–3.9 mm (35.1% (20/57) vs. 22.1% (15/68), *P* = 0.115). At 3-month follow-up, the finial SFR remained comparable between FANS and mini-PCNL (89.5% (51/57) vs. 88.2% (60/68), *P* = 1.000). Complete clearance was achieved in 71.9% (41/57) and 66.2% (45/68) of patients, respectively (*P* = 0.563).

The re-treatment rate showed no difference between two groups (*P* = 0.742). Causes of readmission included ureteroscopic lithotripsy management of residual lower calyx stones (1.8% (1/57) vs. 1.5% (1/68)), and extracorporeal shockwave lithotripsy (3.5% (2/57) vs. 2.9% (2/68)). Analysis of stone composition revealed no statistically significant differences between the groups (*P* = 0.073). calcium oxalate dihydrate (COD) and calcium oxalate monohydrate (COM) were the most common stone types.

### Postoperative complications

Postoperative complications according to the modified Clavien system are summarized in Table 3. The overall complication rate was significantly lower in the FANS group compared with the mini-PCNL group (5.9% (4/57) vs. 25.0% (17/68), *P* = 0.011). There is no difference in Grade 1 complications between two groups (*P* = 0.503), fever > 38 °C occurred in 3 patients (5.3%) in the FANS group and 6 patients (8.8%) in the mini-PCNL group, tube dislodgement was only occurred in 2 patients (2.9%) in the mini-PCNL group. Grade 2 complications were observed only in the mini-PCNL group, including 2 cases (2.9%) requiring blood transfusion and 1 case (1.5%) of heart failure. Perirenal hematoma managed non-operatively was seen in 1 patient (1.7%) in the FANS group and 3 patients (4.4%) in the mini-PCNL group (*P* = 0.122). Grade 3b complications occurred exclusively in the mini-PCNL group, comprising 3 cases (4.4%) of renal hemorrhage requiring angioembolization, 1 case (1.5%) of bladder rupture due to bleeding, and 1 case (1.5%) of septic shock (*P* = 0.062). No Grade 4a–5 complications were reported in either group. Minor (grades 1–2) and major (grades 3–5) complications also were comparable. In summary, although most complications were minor, FANS was associated with a significantly lower overall complication rate and avoided severe events requiring invasive management, compared with mini-PCNL.

**Table 3:** Postoperative complications

Modified Clavien system	FANS (57)	Mini-PCNL (68)		*P* value
Grade 1, n (%)			0.503	
Tube dislodgement	0 (0.0)	2 (2.9)		
Fever (> 38 °C)	3 (5.3)	4 (5.9)		
Grade 2, n (%)			0.122	
Blood transfusion	0 (0.0)	2 (2.9)		
Heart failure	0 (0.0)	1 (1.5)		
Nonoperative of perirenal hematoma	1 (1.7)	3 (4.4)		
Grade 3b, n (%)			0.062	
Renal hemorrhage requiring angioembolization	0 (0.0)	3 (4.4)		
Bladder rupture due to bleeding	0 (0.0)	1 (1.5)		
Septic shock	0 (0.0)	1 (1.5)		
Grade 4a-5, n (%)	0 (0.0)	0 (0.0)	-	
Minor grades 1–2, n (%)	4 (5.9)	12 (17.6)	0.112	
Major grades 3–5, n (%)	0 (0.0)	5 (7.4)	0.058	
Total complication rate, n (%)	4 (5.9)	17 (25.0)	0.011	

## Discussion

Upper urinary tract stones with moderate-to-severe hydronephrosis remain challenging to treat because of distorted anatomy and increased perioperative risks. Traditionally, mini-PCNL has been widely used for stones > 10 mm, given its high stone clearance rates [20]. More recently, FANS has been introduced as a modification of RIRS, aiming to improve irrigation control, visibility, and fragment evacuation, while reducing intrarenal pressure and infectious complications [12]. This has raised the question of whether FANS can provide comparable efficacy but with fewer complications compared to mini-PCNL in this specific population.

### Operative parameters

The FANS group exhibited a significantly longer median operative time compared with mini-PCNL (70.0 vs. 60.0 min, *P* = 0.006). This observation aligns with previous studies suggesting that the introduction of flexible suction-assisted access sheaths and the additional steps required for real-time fragment aspiration may modestly increase procedural duration compared with standard mini-PCNL [21]. The prolonged time reflects the need for repeated sheath repositioning and flexible navigation to reach lower-pole calyces and optimize fragment evacuation, rather than inefficiency of the technique itself.

Despite this moderate increase in operative duration, FANS achieved a smaller hemoglobin drop (8.0 vs. 11.0 g/L, *P* = 0.031), indicating less intraoperative blood loss. The absence of renal parenchymal puncture and tract dilation in FANS eliminates the primary source of vascular injury associated with PCNL. Percutaneous tract creation has been identified as a key determinant of bleeding risk, with larger sheath sizes and multiple tracts independently predicting transfusion and angioembolization requirements [22]. In contrast, retrograde intrarenal access with FANS avoids direct injury to segmental and interlobar arteries, limiting trauma primarily to the urothelial layer.

Moreover, continuous suction during FANS is designed to facilitate a more controlled intrarenal environment with improved fluid evacuation. By potentially maintaining lower intrapelvic pressure, suction-assisted systems may help reduce venous congestion within the collecting system, which could partly contribute to reduced microvascular bleeding. However, it should be emphasized that current evidence regarding intrarenal pressure profiles of suction-assisted systems is largely derived from non-comparative or experimental models, and reported values generally reflect average rather than continuously sustained pressure levels. Therefore, pressure regulation alone is unlikely to fully account for the observed safety differences.

Importantly, in the setting of moderate-to-severe hydronephrosis, renal parenchymal thinning and distortion of the collecting system may increase susceptibility to vascular injury and urinary extravasation during percutaneous access. In such anatomically altered kidneys, the absence of a trans-parenchymal tract may be especially advantageous, independent of intrarenal pressure considerations.

### Renal functional outcomes

There were no significant differences in postoperative eGFR or the drop of eGFR between the FANS and mini-PCNL groups, suggesting comparable short-term preservation of renal function. This finding is consistent with previous clinical and experimental studies indicating that both miniaturized percutaneous systems and flexible retrograde intrarenal approaches exert limited impact on glomerular filtration when performed under controlled intrarenal pressure and with meticulous irrigation management [22, 23].

Although PCNL provides direct collecting system access, it involves renal puncture, which may cause parenchymal injury and transient hemodynamic changes [24]. Mini-PCNL reduces tract size but does not fully eliminate these risks. FANS has been reported to maintain lower average intrapelvic pressure levels (approximately 20–25 mmHg) during the procedure [25, 26]. This reduces pyelovenous backflow and may lower the risk of postoperative kidney injury and infection, helping preserve renal function in patients with hydronephrosis.

Moreover, animal and human imaging studies have shown that both mini-PCNL and flexible ureteroscopy lead only to transient renal parenchymal edema without long-term scarring or cortical thinning, as confirmed by postoperative renal scintigraphy and ultrasound follow-up [23, 27]. Our comparable eGFR drop results further support that both techniques are safe from a renal functional perspective, with FANS potentially offering an additional safety margin due to its non-puncture, low-pressure mechanism.

### SFR and postoperative recovery

In our cohort, immediate SFR at discharge (fragments < 4 mm) and finial SFR at 3 months were comparable between the FANS and mini-PCNL groups (≈ 80% immediate, ≈ 89% at 3 months). These findings suggest that, for stones of moderate size (median maximal diameter ≈ 20 mm), FANS can achieve stone clearance rates equivalent to those of mini-PCNL. The efficacy of FANS in achieving comparable SFR is likely attributable to the combination of flexible navigation, direct fragment aspiration, and dusting strategies, which allow access to lower-pole calyces while minimizing fragment migration [28].

This observation is consistent with recent multicenter analyses and randomized trials demonstrating non-inferiority of suction-assisted flexible ureteroscopy compared with mini-PCNL in stones ranging from 10 to 30 mm. For example, suction-assisted flexible URS achieved SFRs of 80–90% in 20–30 mm stones, comparable to mini-PCNL, while maintaining lower complication rates [28, 29]. These data collectively indicate that, in appropriately selected patients, FANS offers a safe and effective alternative to mini-PCNL without compromising stone clearance.

Patients undergoing FANS had a significantly shorter median hospital stay compared with mini-PCNL (2 vs. 3 days, *P* = 0.008). This likely reflects reduced bleeding, minimal parenchymal trauma, and fewer complications with trans-ureteral, suction-assisted approaches. Recent studies corroborate these findings, reporting shorter hospitalization and faster recovery with suction-enabled flexible ureteroscopy compared with mini-PCNL, without compromising stone clearance [30, 31]. These results suggest that FANS not only maintains efficacy but also enhances perioperative recovery, offering a patient-friendly alternative for moderate-to-large renal stones.

### Complications

The overall complication rate was significantly lower in the FANS group compared with the mini-PCNL group (5.9% vs. 25.0%, *P* = 0.011). Notably, all major complications (Clavien–Dindo grade ≥ 3), including renal hemorrhage requiring angioembolization, bladder rupture, and septic shock, occurred exclusively in the mini-PCNL cohort. Which may suggest a potential safety advantage in endourological stone management. The percutaneous tract formation in PCNL, even with miniaturized access, involves direct renal parenchymal puncture and tract dilation, which can injure segmental vessels and increase the likelihood of bleeding, transfusion, or adjacent organ injury [32]. Severe hydronephrosis was significantly associated with longer operative time, lower stone-free rates, and correlated with increased risk of bleeding and transfusion during minimally invasive PCNL. Severe hydronephrosis is an influential factor that should not be ignored when performing PCNL [33]. Furthermore, severe hydronephrosis itself may predispose to greater procedural vulnerability. Thinning of the renal cortex and distortion of calyceal anatomy can increase the technical complexity of tract establishment and heighten the risk of vascular or collecting system injury. In this context, the transureteral nature of FANS may mitigate anatomical vulnerability rather than solely relying on pressure-related mechanisms.

In contrast, FANS avoids parenchymal puncture, operating entirely through the natural ureteral route. The incorporation of an active suction system is designed to facilitate pressure regulation and efficient evacuation of fragments, which may contribute to improved intrarenal pressure control. Elevated intrarenal pressure has been implicated in postoperative fever, sepsis, and bacteremia [3, 4]. The present findings are consistent with recent systematic reviews and prospective trials reporting that suction-assisted flexible ureteroscopy significantly reduces postoperative fever and infectious complications compared with conventional URS or mini-PCNL, without compromising stone-free outcomes [8, 30].

FANS is designed to help maintain a more stable intrarenal environment, which not only facilitates visualization but may contribute to improved intrarenal pressure regulation. Elevated intrarenal pressure during PCNL has been associated with pyelovenous and pyelolymphatic backflow, potentially leading to bacteremia and postoperative sepsis. The suction-assisted drainage in FANS may theoretically reduce endotoxin absorption and systemic inflammatory responses, which could partly explain the favorable safety profile observed in our study [25, 26].

Taken together, the observed safety profile of FANS in this study is likely multifactorial, reflecting both anatomical preservation and procedural characteristics rather than pressure modulation alone. FANS derives from its minimally invasive transureteral nature and the hemodynamic stability afforded by active suction and pressure-controlled irrigation. For patients with moderate-to-severe hydronephrosis or infection-prone stones, this via ureter and pressure-regulated approach may provide a meaningful safety advantage over percutaneous procedures.

Limitations include single-center sample size, the retrospective design, and the relatively short follow-up (3 months), which limit assessment of long-term recurrence and late complications, in addition, no prospective power calculation was performed due to the retrospective design. Therefore, the study may be underpowered to detect small differences, particularly in subgroup analyses or borderline statistical comparisons. Patient-reported recovery outcomes were not available in this retrospective cohort. Future prospective studies should incorporate standardized patient-reported metrics to enhance comparability across healthcare systems. Well-powered multicenter randomized trials comparing FANS-assisted flexible URS and mini-PCNL across different stone sizes and hydronephrosis grades, with extended follow-up for recurrence, renal function, and cost-effectiveness analyses should be included.

## Conclusions

In this study of patients with moderate to severe hydronephrosis and renal stones with a median maximal diameter of approximately 20 mm, FANS achieved SFR comparable to those of mini-PCNL both at discharge and at 3 months follow-up. Importantly, FANS was associated with several perioperative advantages, including reduced hemoglobin loss, shorter hospital stay, and a lower overall complication rate. These findings indicate that FANS represents a safe and effective minimally invasive alternative to mini-PCNL, offering renal parenchyma preservation and improved perioperative recovery in appropriately selected patients.

## Data Availability

The datasets used and/or analysed during the current study are available from the corresponding author on reasonable request.

## Abbreviations

FNAS: Flexible negative-pressure ureteral sheath lithotripsy: PCNL: Percutaneous nephrolithotomy, OR: operative, WBC: white blood cell, N: number, SFR: stone-free rate, BMI: body mass index, eGFR: estimated glomerular filtration rate, FURS: flexible ureteroscopy, ESWL: extracorporeal shock wave lithotripsy, COD, calcium oxalate dihydrate
COM: Calcium oxalate monohydrate
CP: Calcium phosphate
CC: Cystine calculus
UA: Uric acid
